# Effects of Two Hormonal Protocols for FTAI on the Fertility of Repeat Cows

**DOI:** 10.3390/ijms26125499

**Published:** 2025-06-08

**Authors:** Luis Miguel Vargas Ortiz, Verónica Cristina Andrade Yucailla, Juan Ramón García Díaz, Néstor Vicente Acosta Lozano, Ramón Gonzalo Aragadvay Yungán, Raciel Lima Orozco

**Affiliations:** 1 Facultad de Ciencias Agropecuarias, Universidad Técnica de Ambato, Sector el Tambo-La Universidad, Vía a Quero, Cevallos 1801334, Ecuador; lm.vargas@uta.edu.ec (L.M.V.O.); rg.aragadvay@uta.edu.ec (R.G.A.Y.); 2 Centro de Investigaciones Agropecuarias, Facultad de Ciencias Agropecuarias, Universidad Estatal Península de Santa, km 1 1/2 Vía a Santa Elena, La Libertad 240204, Ecuador; vandrade@upse.edu.ec (V.C.A.Y.); nacosta@upse.edu.ec (N.V.A.L.); 3 Departamento de Medicina Veterinaria y Zootecnia, Facultad de Ciencias Agropecuarias, Universidad Central “Marta Abreu” de Las Villas, Carretera a Camajuaní km 5.5, Santa Clara 54830, Cuba; juanramon@uclv.edu.cu; 4 Centro de Investigaciones Agropecuarias, Facultad de Ciencias Agropecuarias, Universidad Central “Marta Abreu” de Las Villas, Carretera a Camajuaní km 5.5, Santa Clara 54830, Cuba

**Keywords:** repeat cow syndrome, hormone protocols, pregnancy rate, artificial insemination

## Abstract

This study, carried out from January to July 2022 in three provinces of Ecuador, aimed to evaluate the effect of two hormonal protocols for fixed-time artificial insemination (FTAI) on the follicular dynamics, hormonal profile and fertility of dairy cows affected by repeat cow syndrome (RCS). Two groups of Holstein cows with RCS were formed, G1 (conventional) and G2 (J-Sinch), with 26 and 24 animals, respectively. Gynaecological examinations and hormonal determinations in blood serum were carried out. Follicular diameter and concentrations of FSH, LH and P4 were compared by *t*-Student test for independent samples, estrus and pregnancy were compared by binomial comparison of proportions, and factors associated with pregnancy were determined by a model of logistic regression (LR). In G1, the diameter of the dominant follicle was greater (*p* < 0.05) in the left ovary on day 7 following intravaginal device implantation. However, it was similar (*p* > 0.05) in the right ovary on days 7, 8 and 9. The estradiol and LH concentrations at the time of FTAI and the P4 concentrations 15 days after FTAI, as well as the pregnancy rate, were higher in G1 (*p* < 0.05). The LR model explained 60.91% of pregnancies (*p* < 0.001), and the concentrations of estradiol, LH and P4 and the absence of estrus at the time of FTAI had an influence on the pregnancy rate (*p* < 0.05). It was concluded that the inclusion of estradiol benzoate increased the dominant follicle diameter and the concentrations of estradiol, LH and P4 and the pregnancy rate at the first FTAI.

## 1. Introduction

Repeat cow syndrome (RCS) affects female cattle that do not conceive after at least three or more artificial inseminations or natural mating, despite having an apparently normal genital tract, being less than 10 years old, having regular cycles of 17–24 days, and to be inseminated naturally or artificially with semen from fertile bulls in a correctly recognised heat [1,2]. 

From a biological point of view, RCS increases open days (OD) by 6 to 7%, although many studies show that it ranges between 10 and 14% in dairy herds; in Cuba the prevalence was 15.97% in Holstein cows and 9.77% in Cuban Siboney cows (5/8 H × 3/8 C) [2]. However, some studies reported an increase in open days between 14 and 24% due to RCS [3].

RCS has biological, environmental and technological causes. The former includes endocrine disorders resulting in inadequate uterine environment, low estrogen concentrations and signs of heat, shorter estrous cycles, delayed ovulation, anovulatory cycles, inadequate progesterone (P4) concentrations and failure of maternal pregnancy detection [2,4,5].

In addition, various risk factors can influence the subfertility observed in RCS, contributing to imbalances and the occurrence of RCS syndrome. These factors include age, parity, body condition, milk yield, environmental conditions (i.e., heat stress), diseases, and imbalances during the peripartum period, among others [6,7]. 

In fact, RCS is caused by imbalances in mineral nutrition, which negatively impact the synthesis, activation, and regulation of sex hormones, especially LH and P4 [8]. Energy deficiencies, as well as rumen and metabolic diseases including acidosis, fatty liver disease, retained placenta and displaced abomasums, also cause this effect [6,9]. Copper (Cu) and zinc (Zn) deficiencies are common in grazing cattle herds and are associated with repeat service [10,11].

Moreover, RCS is primarily caused by uterine infections, particularly subclinical endometritis. These infections affect uterine conditions that are suitable for implantation and embryo survival [12]. Consequently, there are failures to conceive, early embryonic mortality, repeated estrus and artificial insemination [6].

Also, RCS occurs as a result of technological errors during artificial insemination, such as depositing the semen in the wrong place, performing the procedure at the wrong time or handling it in a non-hygienic way [2,13].

Furthermore, cows with RCS have been diagnosed with low pregnancy rates due to endocrine dysfunction with low estradiol levels during estrus, delayed luteinising hormone peak, oocyte senescence, no ovulation, abnormal fertilisation of oocytes and slow rise in P4 concentrations during the early luteal phase [4].

On the other hand, to manage the estrous cycle and alleviate the biological disorders that cause RCS, there are various hormonal protocols that include gonadotropin-releasing hormone (GnRH), exogenous P4 administered intramuscularly or through intravaginal devices that ensure controlled internal release of the drug, estradiol benzoate, prostaglandin (PgF2α) and combinations of these drugs [14].

Hormonal protocols are used for fixed-time artificial insemination (FTAI), which allows acceptable conception rates to be achieved in dairy cows with irregular cycles [14]. They also increase reproductive efficiency by inducing cyclicity in postpartum cows, anestrus cows and cows with reproductive disorders, achieving pregnancy rates of around 50% [15]. 

Hormonal protocols for FTAI are an alternative for females with RCS [16]. However, there are no conclusive studies on the effect of hormonal protocols for FTAI on the reproductive physiology and fertility of dairy cows with RCS.

The aim of this study was to evaluate the effect of two hormonal protocols for FTAI on follicular dynamics, hormonal profile and fertility in dairy cows with RCS.

## 2. Results and Discussion

Table 1 shows the parameters of follicular dynamics; note that in G1, the diameter of the dominant follicle in the left ovary was greater (*p* < 0.05) on day 7 after IVD implantation compared to G2. The same occurred in the right ovary on days 7, 8 and 9 after IVD implantation. On the other hand, the number of follicles per ovary did not differ between groups (*p* > 0.05).

In G1, the dominant follicle in the left ovary on day 7 was 2.01 mm larger in diameter than in G2, so the left gonad had a higher growth rate in G1 than in G2 (0.98 and 0.69 mm/day, respectively).

In addition, the dominant follicle diameter in the right ovary on days 7, 8 and 9 was 2.38, 0.63 and 1.99 mm larger in G1 than in G2. In G1, the dominant follicle in the right gonad had a higher daily growth rate in G2, reaching values of 1.31 vs. 1.07, 1.17 vs. 1.09 and 1.22 vs. 1.00 mm/day on days 7, 8 and 9 of the protocols started, in the same order.

The dominant follicle diameter and the daily growth rate of the dominant follicle confirmed that the protocols used synchronised follicular growth. BE_2_, administered on day zero, causes atresia of existing follicles and induces, controls and synchronises the emergence of a new wave of follicular growth three to five days after BE_2_ application [17].

The higher daily growth rate of the dominant follicle in G1 may have been influenced by the fact that cows in G1 had a higher CC than those in G2, 3.54 vs. 3.30. In addition, the increase is more pronounced on day 9 because in G1, 1 mg BE_2_ was administered on day 8, 24 h after IVD withdrawal. Exogenous estradiol potentiated the effects of endogenous estradiol and increased the concentration of this hormone in the blood serum. As a result, the negative feedback from inhibin to FSH was favoured, suppressing FSH release and instead releasing LH, which causes increased follicle growth and maturation [18,19].

In this experiment, the diameter of the dominant follicle in the right ovary in G1, on days 7 and 8, was similar to the 9.8 ± 0.5 mm published by Kim et al. [20,21] using BE_2_ in females. The above confirms the importance of administering this hormone in estrus induction and synchronisation protocols or in FTAI.

The daily follicular growth rate of both ovaries in this study is lower than that published by Ginther et al. [22]. This author found a dominant follicle growth rate of 1.5 ± 0.1 and 1.8 ± 0.1 mm/day in the wet and dry seasons, respectively, with no statistical difference between the two seasons, and classified it as normal in cattle. Furthermore, the daily growth rates of the dominant follicles in this experiment are also lower than those published by Pérez G et al. [23] and Alfaro-Astorima et al. [24]. These authors found in lactating and estrus-synchronised cows in the Peruvian highlands that the dominant follicle grew from 2.3 ± 1.5 to 2.4 ± 1.8 mm/day.

The lack of correspondence regarding the daily growth rate of the dominant follicle with that published by Ginther, Baldrighi, Siddiqui, Bashir and Rakesh [22], Pérez G, Quispe B, Luque M, Rojas E, Condori C, Delgado C and Pérez D [23] and Alfaro-Astorima, Ormachea-Sánchez and Alvarado-Malca [24] could be due to differences in rainfall, photoperiod, concentration of plant secondary metabolites and ambient temperature where the research was developed, as these climatic factors affect follicular dynamics [25,26].

Table 2 shows the hormonal profile of the cows and the estrus and pregnancy rates. Note that estradiol, LH and P4 were higher in G1, which may be due to the BE_2_ administered on day 8. This increased their concentrations in the blood serum, which favoured the pre-ovulatory peak of LH, and this increased the production of P4 by the LC and the consequent increase in blood serum P4. It has been shown that the increase in serum estradiol concentrations at the end of the cycle favours the manifestation of estrus, ovulation, the formation of the LC and its production of P4 [27].

BE_2_ administered on day zero causes atresia of existing follicles and induces the emergence of a new follicular wave three to five days after BE_2_ application, ensuring the presence of a new E_2_-producing follicle and a viable oocyte at the end of treatment. BE_2_ also controls and synchronises the follicular growth wave [17].

The higher blood serum LH concentrations in G1 are justified because cows in G1 were administered BE_2_ on day eight of the protocol. This causes an estradiol peak, which has a positive feedback on the release of GnRH by the hypothalamus and consequently increases LH pulses and frequency, thus synchronising estrus presentation and ovulation [18,29].

PgF2α administered on the seventh day of the protocol synchronised estrus and ovulation as a result of LC lysis, which decreased serum P4 concentrations and, through negative feedback on the hypothalamus, induced GnRH release with subsequent release of hypophysial FSH and LH [29].

The higher pregnancy rate achieved in G1 is due to the higher blood serum concentrations of estradiol, LH and P4 in G1 (Table 2), which must have resulted in a higher ovulation rate in cows of this group.

The pregnancy rate achieved in G1, in which the protocol with BE_2_ was applied on day 8 of the start of the protocol, is 26.6% higher than that published by Toro García et al. [30], who diagnosed 44.9% and 53.4% of BE_2_-treated females as pregnant. The reason for the differences between the studies may be due to the animal category, as they worked with heifers, whereas in this experiment, we worked with cows.

The pregnancy rate in this experiment, the group that applied the J-Synch protocol, was lower than that obtained in other studies with the same treatment (i.e. 51% by De la Mata et al. [31] and 52.1% by Yánez-Avalos et al. [32], which may be due to the differences in the breed used in the studies.

The eCG induces a lengthening of the proestrus, which simultaneously generates higher serum concentrations of estradiol produced by the dominant follicle, which favours follicular maturation and improves fertility [33,34].

Table 3 shows the estimated logistic regression (LR) model using maximum likelihood, which explains 60.91% of the pregnancies (*p* < 0.001). Blood serum estradiol and LH concentrations at the time of FTAI, P4 concentrations 15 days after FTAI and the absence of estrus at the time of FTAI had a significant influence on the probability of pregnancy (*p* < 0.05).

The estimated coefficients (Table 3) indicate that the natural logarithm of the probabilities of positive pregnancy increase by 0.01, 8.56 and 1.37 times, respectively, when cows are given 1 IU of estradiol and LH and 1 ng/mL of P4 in blood serum; in addition, they decrease by 3.19 times when there is no estrus at the moment of performing the FTAI.

The OR values (Table 3) show that increasing the concentrations of estradiol and LH in the blood serum at the time of FTAI and of P4 15 days after FTAI increases the probability of pregnancy by 5.34, 52.31 and 3.95 times, respectively. On the other hand, the absence of estrus at the time of the FTAI reduces the probability of pregnancy by a factor of 22.30 times.

The pregnancy rates differed between the protocols applied (*p* < 0.05), so they would be conditioned by hormonal factors and the behaviour of the cows in the presence of estrus symptoms, which coexist and are determinants of the result of the FTAI. For these reasons, LR analysis is a very valuable tool in this type of study [35].

The LR is particularly useful when the response variable is dichotomous (i.e. the probability of pregnancy). The LR expresses the probability of a pregnancy occurring as a function of a set of independent variables, with the aim of describing the event, seeking its causal explanations and a model for predicting it [36].

The increase in the estradiol cascade induced by the administration of BE_2_ on the eighth day of the G1 protocol favours the appearance of estrus signs and the physiological triggering of the preovulatory LH peak, ovulation and the increase in P4 [27]. These authors [27] suggest that blood serum estradiol concentrations remain stable throughout the estrous cycle, and a progressive increase at the end of the cycle is compatible with the mechanism of ovulation and estrus presentation.

In lactating cows, energy requirements are higher than in non-lactating cows. The high feed intake leads to an increase in hepatic blood flow, and then to an increase in the metabolism of estradiol and P4 [37].

Some research associates repeat cow syndrome with endocrine dysfunction in cattle, including higher P4 levels combined with low estradiol concentrations during estrus. In this situation, LH pulses and preovulatory peaks are delayed, resulting in oocyte senescence, impaired ovulation and abnormal fertilisation. Consequently, a slow rise in P4 levels during the luteal phase is associated with low pregnancy rates and early embryonic death [38].

Cows that did not show estrus when inseminated at a fixed time were 22.30 times less likely to become pregnant (Table 3) than cows that showed estrus because the latter would have higher estradiol, LH and P4 levels. In a study by Yánez-Avalos, Barbona, López-Parra and Roberto Marini [32] using the J-Synch protocol, the pregnancy rate in estrus cows was 55% compared to 49% in non-estrus cows. 

There is a direct relationship between the presence of the described clinical signs of estrus and the pregnancy rate, whether the estrus is natural or induced, and whether artificial insemination is performed by conventional methods. However, when estradiol esters are used at the end of hormonal treatments, this behaviour is not the same, since the signs are basically increased by the effect of exogenous estradiol and not by the presence of a preovulatory follicle [39].

In folliculogenesis, there are physiological mechanisms involved in the process of follicular redirection and selection that are not fully understood, but there is a relationship with the receptors for LH in the granulosa of the dominant follicle and an increase in estradiol production by the latter and a decrease in FSH concentrations. Follicular growth also involves insulin-like growth factors (IGF-1 and IGF-2), binding proteins (IGFBP-1, -2, -3, -4, -5 and -6) and specific proteases that degrade and have endocrine, paracrine and autocrine relationships to this process [40].

Pitaluga et al. [41], in FTAI protocols, found a higher proportion of cows in estrus, a better ovulatory response and a tendency to improve corpus luteum diameter during the early luteal phase in cows in which the proestrus period was manipulated with gonadotrophins (eCG) and estradiol.

The hormonal protocols used in FTAI programmes make it possible to concentrate the signs of estrus and ovulation in a given period of time. The use of estradiol salts after withdrawal of the P4 device has the function of inducing a positive feedback on the secretion of gonadotropin-releasing hormone and consequently on LH, in order to induce ovulation in a shorter period of time [30].

## 3. Materials and Methods

The experiment was carried out from January to July 2022 on the farms listed in Table 4.

The study area has a temperate and cold climate, with an average annual temperature of 10 to 15 °C and annual rainfall of 600 to 1900 mm. The average annual rainfall in the study area is between 1000 and 1250 mm. The soil of the study area is of the Andisol type, with an effective depth of 70 cm. The relief is hilly, with a slope percentage that varies between 12 and 25% [42], as determined by GPS map 60 CSx (Garmin, Olathe, KS, USA). The animals in the herd were grazing, and 150 g animal-1 of Calfosal (Multisalmin SA, Quito, Ecuador) was offered ad libitum.

In the herds studied, the grazing system was rationed by electric fencing, with an average global stocking rate of 2.5 UGM ha^−1^. Grazing was carried out for 18 h a day on improved pastures of *Lolium eterna* L., *Trifolium repens* L. and naturalised pastures of *Pennisetum clandestinum* L. and *Cichorium intybus* L. The intake and chemical composition of the pastures are shown in Table 5. Manual milking was performed twice a day, from 2:00 to 4:00 h and from 14:00 to 16:00 h.

A total of 50 Holstein Friesian cows were selected, aged 6.32 ± 1.11 years, with 3.54 ± 0.90 calvings and 3.42 ± 0.33 body condition score on the five-point scale. All cows showed RCS with 4.46 ± 1.35 services per pregnancy. 

The cows were divided into two groups, one (G1; conventional protocol) and two (G2; J-Sinch protocol) of 26 and 24 animals, respectively, whose characteristics are shown in Table 5, and each of them was administered a hormonal protocol for FTAI and the follicular dynamics, hormonal profile and fertility of the cows were compared between them.

All animals were vaccinated against bovine viral diarrhoea, infectious bovine rhinotracheitis, bovine respiratory syncytial virus, parainfluenza and leptospirosis; and clinically healthy, without clinical signs of metritis or pyometra, as diagnosed by the functional invariants of the clinical method [43]. In addition, ovarian activity was diagnosed by ultrasound, mainly the presence of pre-ovulatory follicles and corpora lutea.

### 3.1. Methodologies Used

#### 3.1.1. Body Condition Score Assessment

Body condition score (BCS) was scored on a five-point scale, according to the procedures described by Parker [44].

#### 3.1.2. Hormonal Protocol Diagrams

In both protocols, on day zero, the intravaginal device (DIB^®^ 0.5, Zoetis, Buenos Aires, Argentina) was inserted, and estradiol benzoate (BE_2_; 2 mg, Grafoleon^®^, Life, Quito, Ecuador) was administered. On day seven, the IVD (intravaginal device) was removed, and 0.15 mg of PgF_2α_ (Estrumate, MSD (Merck & Co., Inc.), Kenilworth, NJ, USA) and 400 IU of equine chorionic gonadotropin (eCG; NOVORMON^®^ 5000, Zoetis, Argentina) were administered. 

**Figure 1:**
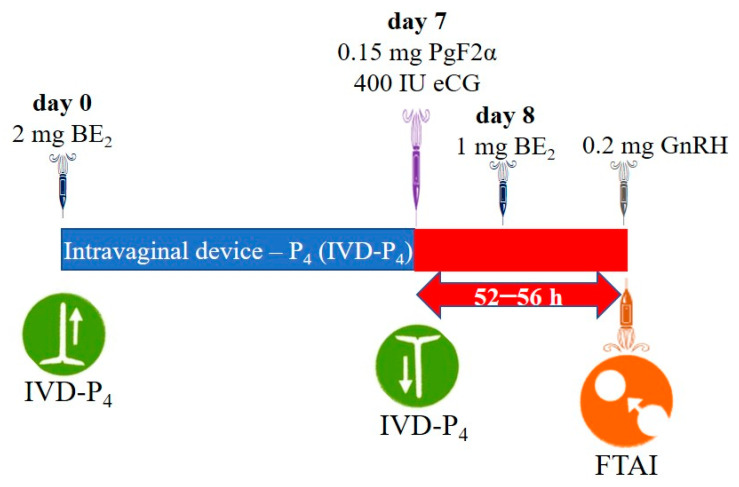
Conventional protocol [45]. BE_2_: estradiol benzoate; eCG: equine chorionic gonadotropin; PgF2α: prostaglandin; GnRH: gonadotropin-releasing hormone; FTAI: fixed-time artificial insemination.

**Figure 2:**
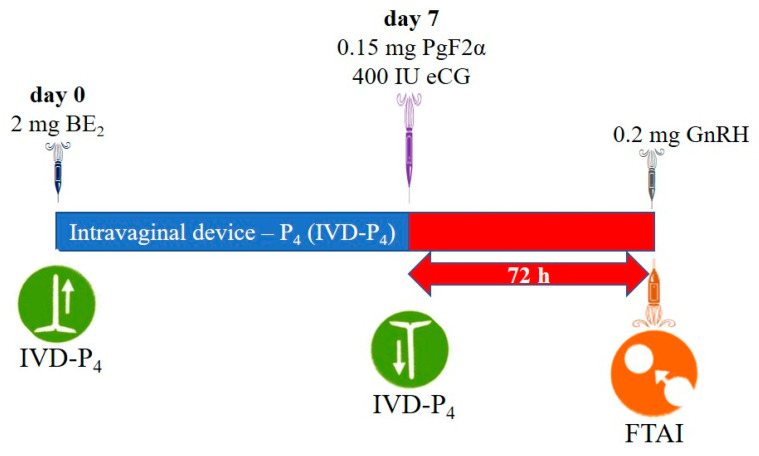
J-Sinch Protocol [46]. BE_2_: estradiol benzoate; eCG: equine chorionic gonadotropin; PgF_2α_: prostaglandin; GnRH: gonadotropin-releasing hormone; FTAI: fixed-time artificial insemination.

In the conventional protocol (G1), BE_2_ (1 mg, Grafoleon^®^, Life, Ecuador) was administered on day 8 and GnRH (0.2 mg Fertagyl, MSD (Merck & Co., Inc.), Kenilworth, NJ, USA) and FTAI were performed 52–56 h after IVD removal, whereas in the J-Sinch (G2), BE_2_ was not administered on day 8 and GnRH (0.2 mg Fertagyl, MSD (Merck & Co., Inc.), Kenilworth, NJ, USA) and FTAI were performed 72 h after IVD removal (Figure 1 and Figure 2). 

#### 3.1.3. Follicular Monitoring and Pregnancy Diagnosis

Gynaecological examinations were performed on days 7, 8 and 9 after implantation of progesterone (P4)-releasing IVDs using an ultrasound scanner (KAIXIN-RKU10, SIUI, China) with a 7.5 MHz linear probe according to the recommendations of Quintela et al. [47] and Sugiura et al. [48]. Follicles were followed until ovulation or atresia, and luteolysis was also performed according to the procedures described by Shrestha et al. [49] and Hannan et al. [50]. All follicles with a diameter ≥ 5 mm were included; the number of corpora lutea and ovarian diameter (OD; average of both ovaries calculated as OD = (length + widest side)/2)) were recorded [48,49], and pregnancy diagnosis was performed at 35 days post-insemination in cows of both groups.

#### 3.1.4. Hormonal Measurements

For hormonal determinations, blood was collected on the day of insemination by puncture of the coccygeal vein and placed in IDEXX VetTube™ tubes (IDEXX LABORATORIES VetLab^®^, Westbrook, ME, USA), which were previously sterilised and demineralised. A 10 mL amount of blood was placed in these tubes without anticoagulant and centrifuged at 3500× *g* for 15 min to obtain blood serum, which was frozen at −10 °C until analysis.

FSH (REF LKFS1), LH (REF LKLH1) and estradiol were determined by the chemiluminescence technique according to the manufacturer’s standards (IMMULITE 1000, Siemens Healthineers, Cary, NC, USA). On the other hand, P_4_ was determined 15 days after artificial insemination by ELISA (Monobind Inc., Lake Forest, CA, USA) using a commercial kit (Progesterone AccuBind ELISA Kit, Monobind Inc., Lake Forest, CA, USA) according to the technical procedures proposed by the manufacturer.

#### 3.1.5. Statistical Processing

Descriptive statistics were performed for each variable. The number and diameter of follicles were compared between the two groups on the different days, and the concentrations of FSH, LH and P4 were compared between the groups using *t*-Student for independent samples, after checking that each variable had a normal distribution and homogeneity of variance. 

Estrus presentation and pregnancy were compared using a binomial comparison of proportions. A logistic regression model using the maximum likelihood method was used to determine the factors associated with pregnancies achieved in inseminated cows. 

The statistical package Statgraphics Centurion Ver. XV.II (Statistical Graphic Corp., The Plains, VA, USA) of 2006 was used for all processing.

## 4. Conclusions

Under the experimental condition of the current study, the inclusion of BE_2_ in hormonal protocols for FTAI, 24 h after removal of the intravaginal device, increases the diameter of the dominant follicle and serum LH concentrations, as well as the pregnancy rate at first FTAI. Therefore, the exogenous application of BE_2_ in FTAI protocols benefits reproductive physiology and improves reproductive indices.

## Figures and Tables

**Table 1:** Number and diameter of follicles (mean ± standard error of the mean) with two hormone protocols.

Variables	Groups	Days		
		7th	8th	9th
Left ovarian follicles, n	1	2.92 ± 0.21 ^a^	2.50 ± 0.21 ^a^	2.53 ± 0.23 ^a^
	2	2.79 ± 0.22 ^a^	2.87 ± 0.23 ^a^	3.08 ± 0.23 ^a^
Dominant follicle diameter in the left ovary, mm	1	6.88 ± 0.64 ^a^	8.23 ± 0.63 ^a^	8.70 ± 0.91 ^a^
	2	4.87 ± 0.67 ^b^	8.29 ± 0.66 ^a^	8.54 ± 0.91 ^a^
Right ovarian follicles, n	1	2.22 ± 0.21 ^a^	2.23 ± 0.23 ^a^	2.26 ± 0.26 ^a^
	2	2.20 ± 0.22 ^a^	2.45 ± 0.20 ^a^	3.00 ± 0.26 ^a^
Dominant follicle diameter in the right ovary, mm	1	9.92 ± 0.70 ^a^	9.42 ± 0.74 ^a^	11.03 ± 0.90 ^a^
	2	7.54 ± 0.72 ^b^	8.79 ± 0.77 ^b^	9.04 ± 0.94 ^b^

^a,b^ Different letters in the superscripts of the values within each column (day) for each variable indicate significant statistical differences for *p* < 0.01 (*t*-Student for independent samples).

**Table 2:** Hormonal profile (x ±EE) and estrus and pregnancy rates with two hormonal protocols.

Variables	Groups	
	G1	G2
FSH (UI)	0.14 ± 0.005 ^a^	0.14 ± 0.006 ^a^
Estradiol (UI)	140.30 ± 12.45 ^a^	110.14 ± 9.12 ^b^
LH (UI)	0.32 ± 0.04 ^a^	0.20 ± 0.01 ^b^
Progesterone (ng/mL)	4.78 ± 0.41 ^a^	4,59 ± 0.39 ^a^
Estrus rates	0.76 ^a^	0.62 ^a^
pregnancy rates	0.76 ^a^	0.41 ^b^

^a,b^ Different superscript letters in the same row indicate statistical differences, *p* < 0.05. FSH, estradiol, LH and progesterone were compared using the *t*-Student test, and estrus and pregnancy rates were compared using a binomial comparison of proportions according to Steel et al. [28].

**Table 3:** Parameters of the estimated maximum likelihood logistic regression model for factors associated with artificial insemination outcomes.

Factors	EC	SE	OR	χ^2^	GL	*p*
Constante	−9.62	4.60	-			
Estradiol in blood serum	0.01	0.01	1.51	5.34	1	0.021
LH in blood serum	8.56	4.61	52.31	6.59	1	0.010
Absence of estrus at time of FTAI	−3.10	1.58	22.30	5.42	1	0.020
P4 concentrations 15 days after FTAI	1.37	0.51	3.95	16.41	1	<0.001
Number of inseminations per protocol	1.56	1.16	4.80	2.06	1	0.150

FTAI: Fixed-Time Insemination; EC: Estimated Coefficient; SE: Standard Error; OR: Odds Ratio; GL: Degrees of Freedom; *p*: Statistical Significance.

**Table 4:** Farms used in the study.

Farm	Location [North Latitude (NL) and West Longitude (WL)]	Height (m.a.s.l.)	Municipality	Province
El Rosario	778,628 and 77″ NL and 987,541 WL	3280	Salcedo	Cotopaxi
Camila	773,358 and 77″ NL and 988,390 WL	3020	Salcedo	Cotopaxi
Planchaloma	747,202″ NL and 9,919,812 WL	3350	Latacunga	Cotopaxi
El Carbón	771,800″ NL and 98,972,723 WL	3200	Pillaro	Tungurahua
Chañag	775,230″ NL and 9,821,015 WL	3228	Riobamba	Chimborazo
Cubijies	775,230″ NL and 9,821,015 WL	3228	Riobamba	Chimborazo

**Table 5:** Bioproductive characteristics of the cows selected in the experiment.

Statistigraph	Age (Years)		Births (n)		AIR (n)		BCS (Points)		OD (Days)		MY (L/Caw^−1^)	
	G1	G2	G1	G2	G1	G2	G1	G2	G1	G2	G1	G2
Mean	6.11	6.54	3.69	3.37	4.38	4.54	3.54	3.3	216.34	239.54	9.61	10.83
SD	1.14	1.06	0.97	0.82	1.32	1.41	0.37	0.23	54.46	67.13	2.22	3.78

n: number; AIR: artificial insemination rate; BCS: body condition score; OD: open days; MY: milk yield; SD: standard deviation.

## Data Availability

The data presented in this paper are available on request from the corresponding author.
